# False Perceptions

**Published:** 1883-04

**Authors:** William A. Hammond


False Perceptions.—The simplest forms of insanity are those which consist merely of false perceptions, and they are not of such a character as to lessen the responsibility of the individual. There are two forms of false perceptions—illusions and hallucinations. Uncomplicated illusions are rare; still there is no doubt that there are illusions not the results of disease in the organs of sense or of circumstances unfavorable to exact perception, but which are due to a morbid condition of the perceptional ganglia, and the unreal nature of which is clearly recognized by the individual.

Illusions of *sight* often relate merely to the size of the objects. Thus, a young lady who had overtasked herself at school saw everything of enormous size at which she looked. The head of a person seemed to be several feet in diameter, and little children looked like giants. So far as her own person was concerned there were no illusions. Her own hands appeared of the natural size, but those of other people seemed to be of enormous proportions. Sauvages refers to a case in which a young woman, suffering from epilepsy, had the illusion of seeing objects greatly magnified. A fly seemed to her to be as large as a chicken. In the case which came under my observation, the unreal character of the perception was fully recognized, and hence the intellect was not involved.

Morbid illusions of *hearing*, unaccompanied by other evidences of mental derangement, are not very common. One case only has come under my observation. It was that of a gentleman to whom the ticking of a clock was resolved into articulate words. Generally the expressions were in the form of commands. For instance, if at dinner, they would be, "Eat your soup!" "Drink no wine!" and so on. One day he made the discovery that, if he closed the right ear firmly, the illusion disappeared; but, if the left ear were closed, the words were still distinctly heard. It was hence clear that the center for hearing on the right side was the one affected, and that that on the left side was normal. For a long time this gentleman resisted accepting any of these illusions as facts, but after a time he began to be influenced by them to the extent of regarding them as guides. Eventually he put clocks in every room in his house, and professed to be governed altogether by the directions they gave him.—Dr. William A. Hammond, *in Popular Science Monthly for April.*

